# The Effects of 8-Week Hydrogen-Rich Water Consumption on Appetite, Body Composition, Sleep Quality, and Circulating Glucagon-like Peptide-1 in Obese Men and Women (HYDRAPPET): A Randomized Controlled Trial

**DOI:** 10.3390/medicina61071299

**Published:** 2025-07-18

**Authors:** Nikola Todorovic, Sonja Baltic, David Nedeljkovic, Jovan Kuzmanovic, Darinka Korovljev, Dejan Javorac, Katarina Bijelic, Nebojsa Kladar, Alex Tarnava, Sergej M. Ostojic

**Affiliations:** 1 Applied Bioenergetics Lab, Faculty of Sport and Physical Education, University of Novi Sad, 21000 Novi Sad, Serbia; nikolatodorovic1708@gmail.com (N.T.); sonjapaljic@gmail.com (S.B.); david.nedeljkovic7@gmail.com (D.N.); kuzman_07@hotmail.com (J.K.); korovljev.darinka@gmail.com (D.K.); javorac.dejan@gmail.com (D.J.); 2 Faculty of Medicine, University of Novi Sad, 21000 Novi Sad, Serbia; katarina.bijelic@mf.uns.ac.rs (K.B.); nebojsa.kladar@mf.uns.ac.rs (N.K.); 3 Natural Wellness Now Health Products Inc., Maple Ridge, BC V4R 2S6, Canada; alextarnava@gmail.com; 4 Faculty of Health Sciences, University of Pecs, 7624 Pecs, Hungary; 5 Department of Nutrition and Public Health, University of Agder, 4604 Kristiansand, Norway

**Keywords:** dihydrogen, cravings, cholesterol, sleep, supplementation, GLP-1

## Abstract

*Background and Objectives:* Preliminary studies indicate that dihydrogen (H_2_) may affect molecular pathways involved in appetite regulation; however, its role in influencing patient-reported appetite outcomes in individuals with obesity remains uncertain. This randomized, placebo-controlled, double-blind trial aimed to evaluate the effects of H_2_ supplementation on appetite, body composition, sleep quality, obesity-specific quality of life, and related biomarkers in obese men and women. *Materials and Methods:* The study included 36 participants (24 females; age 42.1 ± 13.2 years; BMI 30.8 ± 4.2 kg/m^2^) randomized to receive either 1.0 L of hydrogen-rich water (15 mg of H_2_) or 1.0 L of control water (0 mg of H_2_) daily for eight weeks. *Results:* The results demonstrated that hydrogen-rich water significantly mitigated cravings (*p* = 0.05), improved subjective sleep quality (*p* = 0.05), reduced total cholesterol (*p* = 0.02) and LDL cholesterol (*p* = 0.04), and increased plasma glucagon-like peptide-1 levels (*p* = 0.05) compared to the control. No severe adverse effects were reported throughout the trial. *Conclusions:* These findings suggest that hydrogen-rich water may serve as a safe and effective dietary strategy to address appetite regulation and related metabolic indices in individuals with obesity. The study is registered at ClinicalTrials.gov (NCT06722326).

## 1. Introduction

Molecular hydrogen (H_2_, dihydrogen) has emerged as an innovative biomedical agent with significant therapeutic potential across various domains of human health. Among these, dihydrogen shows particular promise for metabolic conditions. Recent studies have demonstrated the benefits of molecular hydrogen in individuals with metabolic syndrome [1,2,3], type 2 diabetes [4,5], non-alcoholic fatty liver disease [6,7,8], hyperuricemia [9], and obesity [10,11]. In these conditions, dihydrogen likely acts as a hormetic and signaling agent. It may also help suppress endoplasmic reticulum stress, activate autophagy, upregulate mitochondrial function, and regulate gut microbiota (for a detailed review, see References [12,13,14]). Preliminary findings suggest that dihydrogen might also influence the cerebral regulation of energy homeostasis, potentially impacting both orexigenic and anorexigenic signaling [15]. Our research group recently demonstrated that dihydrogen can modulate the glutamate–GABA–glutamine cycle, a critical pathway underlying appetite suppression and weight-loss effects [16]. However, the effects of dihydrogen on other key neurotransmitters involved in appetite regulation, such as glucagon-like peptide-1 (GLP-1), and on appetite measures in obese individuals remain unknown. Thus, the primary aim of this randomized controlled trial was to evaluate the effects of dihydrogen supplementation on body composition indices, appetite, obesity-specific quality of life, and circulating GLP-1 levels in obese men and women. We hypothesized that dihydrogen would upregulate GLP-1 levels, reduce appetite, and promote weight loss within this population.

## 2. Materials and Methods

### 2.1. Participants

The present trial employed a parallel-group, randomized, placebo-controlled, double-blind design, with an allocation ratio of 1:1 between the experimental group (hydrogen-rich water) and the control group (placebo). Eligibility criteria for participant inclusion required individuals to be aged 18–65 years, classified as obese (body fat > 30% for women and >25% for men), and sedentary, defined as engaging in less than 150 min of moderate physical activity per week. Exclusion criteria included the presence of any major chronic diseases or acute injuries at the time of recruitment, dietary supplement use within four weeks prior to study initiation, use of obesity-related pharmaceutical within eight weeks prior to study initiation, refusal to consent to randomization, and concurrent participation in other trials. All eligible participants provided informed consent, and ethical approval was obtained from the local IRB at the University of Novi Sad (#50-06-18/2024-1). The study adhered to the principles of the Declaration of Helsinki (7th revision). Data collection took place at the Applied Bioenergetics Lab at the University of Novi Sad between April and December 2024. The baseline characteristics of study participants are presented in Table 1. The study is registered at ClinicalTrials.gov (NCT06722326).

### 2.2. Interventions

Participants in the experimental group received hydrogen-rich water at a daily dosage of 1.0 L, while the control group (placebo) received an equal amount of tap water. Both interventions were administered three times daily (morning, early afternoon, and before dinner; 333 mL per serving) with a dihydrogen concentration of 5 mg per serving in the experimental drink (totaling 15 mg of H_2_ per day) and 0 mg per serving in the control drink. The appearance, texture, and sensory characteristics of both drinks were identical. The interventions were provided by Natural Wellness Now Health Products Inc. (Maple Ridge, BC, Canada). The intervention period lasted eight weeks, during which participants were asked to refrain from using any other nutritional supplements or weight management interventions, including diet modifications, exercise, anti-obesity medications, behavioral therapy, or bariatric surgery.

### 2.3. Outcomes

The study’s predetermined primary and secondary outcomes included appetite assessments, body composition indices, obesity-related quality of life, sleep quality components, biochemical markers, and the prevalence and severity of side effects. The primary endpoint was the change in appetite (total cravings score) from baseline to the follow-up (see below). All measures were assessed at baseline (pre-administration) and at the 8-week follow-up (post-administration). Laboratory assessments were conducted between 08:00 and 12:00 following an overnight fast. Participants were instructed to abstain from physical exercise for 12 h and to avoid alcohol, coffee, tea, fizzy drinks, and energy drinks for 24 h before measurements. Anthropometric measurements included height (Seca 210, Hamburg, Germany) and weight (Omron BF508, Tokyo, Japan), with body mass index (BMI) calculated as weight in kilograms divided by the square of height in meters. Waist circumference was measured with an anthropometric tape (Gulic CHP, Ann Arbor, MI, USA). Body composition was assessed using a multifrequency bioelectrical impedance analyzer (BioScan 920, Maltron International Ltd., Rayleigh, Essex, UK), recording parameters such as fat and fat-free mass, muscle mass, total and compartmental body water (intracellular and extracellular), protein mass, mineral mass, total body calcium, and glycogen mass. Appetite was assessed using the Food Cravings Questionnaire (FCQ) [17], a validated tool designed to measure five key dimensions of appetite: (1) an intense desire to eat, (2) the anticipation of positive reinforcement from eating, (3) the expectation of relief from negative emotional states through eating, (4) a lack of control over eating behaviors, and (5) cravings as a physiological state, such as hunger. Respondents rated items on a 5-point Likert scale ranging from 1 (strongly disagree) to 5 (strongly agree), with higher scores reflecting greater intensity of food cravings. The Impact of Weight on Quality of Life-Lite (IWQOL-Lite) questionnaire [18], a validated 31-item self-report tool, assessed obesity-specific quality of life across five domains: physical function, self-esteem, sexual life, public distress, and work, with scores ranging from 0 to 100 (higher scores indicating better quality of life). Sleep quality was measured using the Pittsburgh Sleep Quality Index (PSQI) [19], covering seven subcategories (subjective sleep quality, sleep latency, sleep duration, sleep efficiency, sleep disturbance, use of sleep medication, and daytime dysfunction), where higher scores indicate more significant sleep disturbances. Fasting blood samples were collected at each lab visit for biochemical analyses. Glucose, total cholesterol, triglycerides, and lipoprotein levels were determined by standard enzymatic methods using an automated analyzer (Hitachi, Tokyo, Japan). Serum levels of short-chain fatty acids (SCFAs)—acetic acid, propionic acid, and butyric acid—were measured by a sensitive gas chromatography tandem mass spectroscopy method with modifications [20]. Plasma GLP-1 levels were assessed with a commercial ELISA kit (Elabscience, Houston, TX, USA). Molecular hydrogen levels in breath were measured using an electrochemical fuel cell microprocessor (LactoFAN2, Fischer Analysen Instrumente GmbH, Leipzig, Germany). Participants were also asked to report any side effects (e.g., stomach upset, bloating, constipation, diarrhea, nausea, or vomiting) experienced due to either intervention throughout the study using an open-ended questionnaire. No modifications to trial outcomes were made after the study began.

### 2.4. Statistical Analyses

The minimum sample size (*n* = 24) was determined by power analysis using G * Power 3.1 (Heinrich-Heine-Universität Düsseldorf), with an effect size of 0.30 (indicating a small effect), an alpha level of 0.05, and a power of 0.80, based on the anticipated change in appetite (total cravings score) from baseline to the 8-week follow-up. This calculation assumed two groups with two measurement points for study outcomes. To allow for potential attrition, the sample size was increased to 36 participants. To maintain balanced participant characteristics across groups, a stratified randomization model was applied, creating separate blocks based on gender (male and female). Data normality was assessed using the Shapiro–Wilk test, and variance homogeneity was examined with Bartlett’s test. Within-group differences over time were compared using *t*-tests for normally distributed data and the Wilcoxon Signed-Ranks Test for non-normally distributed data. For data with a normal distribution and homogeneous variances, interaction effects (time vs. intervention) were analyzed with a mixed-model ANOVA. In cases of non-homogeneous variances, comparisons were conducted using the Friedman test. Effect sizes for within-group comparisons were calculated using Cohen’s *d*, while interaction effects were assessed using partial eta squared (*η_p_*^2^). Given the multiple comparisons across outcomes, we interpreted results with caution, emphasizing consistent patterns and effect sizes rather than relying solely on nominal *p*-values. Formal correction for multiple testing (e.g., Bonferroni correction) was not applied due to the exploratory nature of the study and the relatively small sample size. Statistical significance was set at *p* ≤ 0.05. Missing data were excluded from the analyses. All statistical analyses were conducted using SPSS version 24.0 for Mac (IBM SPSS Statistics, Chicago, IL, USA).

## 3. Results

**Figure 1:**
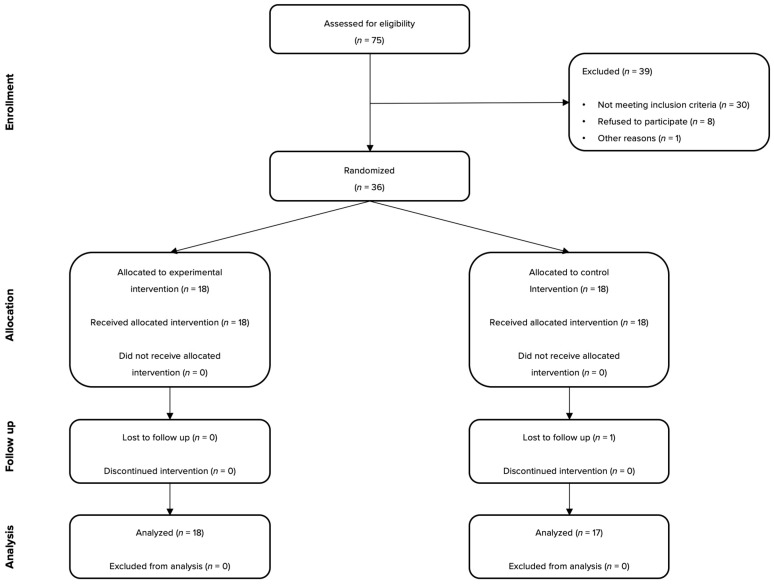
Flow of participants during the study.

A total of 35 participants (*n* = 35; 23 females) were randomly assigned to the intervention or control group, received the allocated supplementation, and were included in the primary outcome analysis. Of these, 18 participants (12 females) were in the experimental group, and 17 participants (11 females) were in the control group. The flow of participants through each stage of the randomized trial is depicted in Figure 1.

Changes in primary and secondary outcomes over the course of the trial are presented in Table 2 (appetite assessments), Table 3 (body composition indices), Table 4 (obesity-related quality of life), Table 5 (sleep quality components), and Table 6 (biochemical markers).

Hydrogen-rich water consumption significantly reduced all dimensions of appetite after eight weeks compared to baseline values in the overall sample, except for the dimension related to a lack of control over eating behaviors. No significant differences were observed in the placebo group when comparing pre- and post-intervention measurements across the entire sample. A significant interaction effect (time × treatment) was observed for cravings as a physiological state (*p* = 0.05) and total scores for cravings (*p* = 0.05), with hydrogen-rich water demonstrating greater efficacy than the placebo in mitigating these specific appetite dimensions in the overall sample. The effect sizes for these interactions exceeded the threshold for large effects (*η_p_*^2^ > 0.14). Gender-specific analyses yielded comparable findings for the female subsample, showing significant interaction effects (with large effect sizes) for cravings as a physiological state and total scores (*p* < 0.05).

Hydrogen-rich water consumption resulted in a significant reduction in body weight among the male subsample (mean change: −2.0 ± 2.4 kg; *p* = 0.05) after 8 weeks compared to baseline values. No significant changes were observed for other body size or body composition indices in the overall sample or within gender-specific subsamples during the pre–post-assessment period. No significant interaction effect was observed between interventions for body size or composition indices throughout the trial. However, a notable trend (*p* = 0.07) suggested that hydrogen-rich water may outperform the placebo in reducing body weight among men.

Hydrogen-rich water consumption significantly improved physical function, self-esteem, sexual life, and total scores for obesity-related quality of life after eight weeks compared to baseline values in the overall sample. The placebo intervention similarly improved self-esteem and cumulative scores in pre–post comparisons within the total sample. Still, no significant interaction effects were observed across the total sample. Subgroup analysis revealed significant improvements across nearly all indices (excluding public distress) following hydrogen-rich water intake, and in physical function and work performance following placebo intake in females, with no significant interaction effects observed between interventions in this group. In males, no significant changes were detected during the trial except for a self-esteem interaction effect (*p* < 0.01), where the placebo demonstrated greater efficacy than hydrogen-rich water in enhancing this variable.

Hydrogen-rich water consumption significantly enhanced subjective sleep quality, sleep latency, sleep disturbance, daytime dysfunction, and total sleep scores post-intervention compared to baseline values in the overall sample. Similarly, the placebo intervention improved subjective sleep quality, sleep latency, sleep duration, daytime dysfunction, and total sleep scores in pre–post comparisons. Notably, a significant interaction effect was observed for subjective sleep quality (*p* = 0.05), with hydrogen-rich water demonstrating superior improvements compared to the placebo across the overall sample. Subgroup analysis identified several domain-specific improvements in sleep following either intervention in pre–post comparisons, with a significant interaction effect (*p* = 0.05) observed for the use of sleep medication in the female subsample.

The consumption of hydrogen-rich water significantly increased serum GLP-1 levels in both the entire sample and the female subsample after 8 weeks compared to baseline. In contrast, no significant changes in serum GLP-1 levels were observed in the placebo group during the pre–post-assessment period. A significant interaction effect was identified for serum GLP-1 levels in the entire sample (*p* = 0.05), indicating that hydrogen-rich water was more effective than the placebo in increasing serum GLP-1 levels, with a large effect size for the interaction (*η_p_*^2^ = 0.20).

The consumption of hydrogen-rich water had no effect on serum acetic acid levels but significantly reduced serum propionic acid and butyric acid concentrations in the overall sample and within both gender subgroups after 8 weeks, compared to baseline. Similarly, the placebo intervention led to reductions in circulating propionic acid and butyric acid in pre–post comparisons. No significant interaction effects between the interventions were observed for any of the three serum SCFAs throughout the trial.

Drinking hydrogen-rich water significantly reduced total cholesterol, LDL cholesterol, and HDL cholesterol levels after eight weeks of administration compared to baseline values, while no significant changes were observed in the placebo group during pre–post comparisons across the overall sample. A significant interaction effect was detected for total cholesterol (*p* = 0.02) and LDL cholesterol (*p* = 0.04), with hydrogen-rich water showing superior reductions in these parameters compared to the placebo. Subgroup analysis revealed notable improvements in lipid profiles following hydrogen-rich water consumption, with a significant interaction effect observed for total cholesterol in the female subsample (*p* = 0.04). The effect sizes for above interactions were considered large (*η_p_*^2^ > 0.14).

Finally, no participants reported any severe adverse effects that impeded their participation in the trial; one participant was lost to follow-up. Among those in the experimental group, one female participant (aged 50) noted more frequent bowel movements after the intervention, while another female participant (aged 51) reported a reduction in dizziness frequently experienced prior to the study. In the control group, one female participant (aged 25) indicated an improvement in work performance during daily activities. Adherence to the intervention was high, averaging 98.3 ± 2.4% in the experimental group and 97.1 ± 2.9% in the control group (*p* = 0.18), based on the number of unused bottles.

## 4. Discussion

Our trial is among the first to evaluate the effects of hydrogen-rich water on appetite-related indicators and associated outcomes in individuals with obesity. The findings revealed that hydrogen-rich water, administered over an eight-week period, was superior to a placebo in reducing food cravings, lowering serum total and LDL cholesterol levels, and upregulating GLP-1 levels in our cohort of obese participants. These effects were particularly pronounced in women, with hydrogen-rich water demonstrating large effect sizes for these outcomes. Additionally, hydrogen-rich water outperformed the placebo in improving subjective sleep quality. No adverse effects were reported, and no major differences in body composition or obesity-related quality of life measures were observed between interventions during the study period. These results suggest that hydrogen-rich water is a safe and potentially effective dietary intervention for reducing appetite and improving lipid profiles in adults with obesity. Further research is warranted to explore these effects in larger and more diverse populations.

A limited number of small-scale studies have investigated the potential effects of dihydrogen on appetite and related mediators within experimental and clinical nutrition contexts. A Japanese study was among the first to show that a 4-day supplementation with hydrogen water can influence mRNA expression for ghrelin in mice [21], an appetite-stimulating and glucose-regulating hormone that plays a key role in increasing caloric intake and fat deposition. Similarly, another animal study indicated that hydrogen-rich water, consumed over 25 days, could influence daily weight gain, feed intake, and upregulate serum levels of appetite-regulating hormones such as peptide YY and cholecystokinin in female-only piglets fed a mycotoxin-contaminated diet, compared to a control group [22]. Furthermore, a 12-week hydrogen-rich water intervention in five overweight women demonstrated effects on the brain’s glutamate–glutamine–GABA cycle, which involves critical amino acid neurotransmitters in neural activation related to appetite regulation [16]. A recent human study explored hydrogen-related appetite control pathways in eight patients (one male and seven female) with obesity who had undergone Roux-en-Y gastric bypass (RYGB) surgery [23]. The authors found that a single dose of a hydrogen-producing compound (inulin) acutely enhanced the glucose-lowering and appetite-suppressive effects of surgery, correlating with breath hydrogen concentrations, though with no measurable effects on plasma GLP-1 and peptide YY. Our study corroborates some findings from these prior investigations, demonstrating positive effects of hydrogen-rich water on appetite markers and lipid profiles. It also expands on these findings by utilizing a longer supplementation period, a larger and gender-diverse sample, and a more comprehensive evaluation of appetite and body composition, focusing specifically on obese individuals.

Our primary finding indicates that hydrogen-rich water was significantly more effective than a placebo in suppressing appetite, evidenced by a reduction of 7.4 points in the total cravings score in the experimental group compared to 1.3 points in the control group. This suggests that dihydrogen may act as an appetite suppressant, particularly in individuals with obesity. The effect was more pronounced in the female subsample, likely targeting physiological mechanisms underlying cravings, as reflected by a significant intervention-specific difference in this appetite subdomain. Dihydrogen may suppress appetite in individuals with obesity through several interconnected physiological and biochemical pathways. It has been proposed to modulate gut hormones, such as ghrelin, and gut-derived metabolites like SCFAs, which are involved in appetite regulation [16,24]. Additionally, dihydrogen may influence appetite-related brain regions and neurotransmitter systems, contributing to its effects on central appetite control [15,16]. By mitigating oxidative stress and inflammation—key factors in dysregulated appetite observed in obesity—dihydrogen could normalize hunger and satiety signals [25]. Furthermore, it may address insulin resistance, which is known to impair the regulation of hunger and fullness [12], and alter fatty acid availability, which is closely linked to appetite control [26]. H_2_ may also enhance hydration levels, independently promoting a sense of fullness and thereby reducing caloric intake [27]. Lastly, dihydrogen may influence brain estrogen levels, which are implicated in appetite regulation, as demonstrated in an animal study [28]. The findings indicate that these effects are more pronounced in females, suggesting a potential gender-specific mechanism of action. Our previous study highlights the potential involvement of gut-derived SCFAs in appetite regulation, with hydrogen-rich water significantly elevating fecal propionic levels in individuals with obesity [16]. SCFAs, produced through colonic fermentation, are known to activate hormonal and neural pathways that suppress appetite and reduce energy intake [29]. However, the mechanisms by which hydrogen-induced SCFA production in the gut translates into systemic circulation and influences the brain to regulate appetite remain poorly understood. The present study revealed a reduction in circulating levels of propionic and butyric acid following hydrogen-rich water consumption, with the effects similar to the placebo. This decrease in gut-derived propionic acid may result from its utilization by the liver through first-pass metabolism [30] or potentially increased uptake by other tissues, including the brain [31]. Recent studies have highlighted the intricate interplay between the fecal microbiota and plasma metabolites following hydrogen intervention [32,33], underscoring the need for further research to elucidate the liberation, absorption, distribution, metabolism, and clearance of endogenous SCFAs after hydrogen intake. However, hydrogen-rich water appears to influence GLP-1, a key hormone in appetite regulation. Our trial observed mild-to-moderate increases in serum GLP-1 levels following hydrogen-rich water consumption. GLP-1 plays a critical role in managing appetite by acting on gastrointestinal and brain satiety pathways [34]. These findings suggest that hydrogen-rich water may serve as a novel dietary intervention, potentially modulating GLP-1 metabolism through mechanisms affecting its secretion, cellular uptake, or elimination. The observed increase in GLP-1 levels following hydrogen-rich water supplementation may be mediated by several physiological mechanisms. HRW has demonstrated antioxidant and anti-inflammatory properties, which are known to positively influence enteroendocrine function, including L-cell stimulation and GLP-1 secretion [35]. Emerging evidence suggests that oxidative stress can impair GLP-1 synthesis and secretion, and that redox modulation may restore or enhance this pathway [36]. Furthermore, HRW may affect the gut–brain axis via alterations in gut microbiota and short-chain fatty acid (SCFA) production, both of which are implicated in the regulation of GLP-1 and appetite signaling [15]. Although our study did not show significant increases in SCFA levels, prior research indicates that hydrogen can modulate colonic fermentation and gut hormone output indirectly [16]. Further clinical research is essential to elucidate these pathways and confirm the effectiveness of hydrogen-rich water in appetite suppression among individuals with obesity.

No significant differences in body size or composition between interventions were observed in the present study, likely attributable to the relatively short duration of the intervention. Previous research has indicated a trend toward weight reduction in individuals with non-alcoholic fatty liver disease following 8 weeks of hydrogen-rich water supplementation [7]. However, extended supplementation with hydrogen-rich water, ranging from 12 to 24 weeks, has been demonstrated to reduce body weight in overweight individuals [11] and patients with metabolic syndrome [3]. This suggests that achieving significant weight-related changes with hydrogen-based interventions may require a longer treatment period. However, our findings revealed a notable reduction in body weight (2.0 kg on average) in obese men who consumed hydrogen-rich water, indicating a potential weight-reducing effect specific to this subgroup. Prior studies have shown that men tend to lose weight more rapidly than women after dietary interventions, primarily due to differences in body composition, energy expenditure, and hormonal factors influencing metabolism [37,38]. These physiological and metabolic differences may explain the observed male-specific response to hydrogen-rich water. Although the magnitude of this effect appears modest, the possibility of a gender-specific response to hydrogen highlights the importance of incorporating sex as a biological variable in future research. Further investigation is warranted to explore the mechanisms underlying these effects and to optimize intervention strategies for different populations.

We observed significant lipid-lowering effects of hydrogen-rich water, evidenced by reductions in total cholesterol and LDL cholesterol levels in individuals with obesity. Specifically, total cholesterol decreased by an average of 0.32 mmol/L (95% CI, from 0.10 to 0.54), while LDL cholesterol declined by 0.21 mmol/L (95% CI, from 0.02 to 0.54) following eight weeks of hydrogen-rich water consumption, outperforming the placebo in modulating these metabolic biomarkers. These findings align with prior studies demonstrating the cholesterol-reducing potential of dihydrogen across diverse clinical populations (for a comprehensive review, see ref. [39]). Although the underlying mechanisms remain to be fully elucidated, it is hypothesized that hydrogen-rich water may exert lipid-lowering effects through antioxidative and anti-inflammatory pathways, modulation of lipid metabolism, or improved insulin sensitivity. These promising results suggest that hydrogen-rich water could serve as a safe and effective dietary supplement for managing dyslipidemia in obesity, warranting further investigation in larger, long-term studies.

Our findings also reveal that hydrogen-rich water significantly improved sleep quality compared to the placebo, with a large interaction effect observed in our cohort of individuals with obesity (*η_p_*^2^ = 0.21). Given that sleep quality is frequently compromised in individuals with obesity—due to interrelated physiological, metabolic, and psychological factors [40]—our data suggest that hydrogen-rich water may represent a novel therapeutic option for addressing sleep disturbances in this population. Emerging evidence aligns with our results, supporting the potential role of dihydrogen in modulating sleep-related outcomes. Preclinical research indicates that dihydrogen can enhance sleep architecture and consolidation through the activation of neuronal pathways in brain regions involved in sleep promotion [41]. Furthermore, a recent clinical study demonstrated that hydrogen gas inhalation improved total sleep duration, sleep efficiency, and reduced sleep latency in patients with glioma, positioning hydrogen as a potential therapeutic agent in sleep medicine [42]. Hydrogen’s potent antioxidant and anti-inflammatory properties may underlie these effects by alleviating oxidative and inflammatory stress, which are known disruptors of circadian rhythms and the sleep–wake cycle, particularly under conditions of physiological or psychological stress [43]. These properties may be particularly beneficial in the context of obesity, where chronic inflammation and oxidative stress are prevalent. While these findings underscore the promising utility of dihydrogen in improving sleep quality, additional research is necessary to clarify the mechanisms involved and to validate its clinical efficacy, particularly in populations with conditions that compromise sleep health.

While our study demonstrated several statistically significant outcomes, it is essential to consider whether these changes are also clinically meaningful. The observed reduction in total food cravings score (−7.4 points) in the hydrogen-rich water group corresponds to a moderate-to-large effect size and exceeds the threshold typically considered clinically relevant in behavioral appetite studies [44]. Similarly, the reductions in total cholesterol (−0.32 mmol/L) and LDL cholesterol (−0.21 mmol/L) fall within the range associated with a modest but meaningful reduction in cardiovascular risk in obese populations, as supported by meta-analyses linking small lipid changes to improved health outcomes [45]. The improvement in sleep quality, with a large effect size, is particularly notable given the high prevalence of sleep disturbances in obesity and the known impact of poor sleep on metabolic and psychological health. Although the increase in GLP-1 levels was moderate, GLP-1 is a clinically validated target in obesity treatment, and even small elevations may contribute to appetite suppression and glycemic control. Together, these findings suggest that the physiological changes observed in our study are not only statistically significant but also potentially clinically meaningful, especially when considering the cumulative impact on obesity-related risk factors.

While the study design demonstrates methodological rigor, several limitations should be acknowledged. The relatively small sample size restricts statistical power and limits the generalizability of the findings, underscoring the need for larger, more diverse cohorts to validate these results across broader populations. Self-reported questionnaires for assessing appetite, sleep, and quality of life introduce potential biases, including social desirability and recall inaccuracies, which may affect the reliability of subjective outcomes. Despite instructions to avoid additional dietary supplements and weight management interventions, the absence of formal dietary intake and physical activity monitoring presents a risk of confounding variables influencing the results. The 8-week intervention period, while sufficient for initial assessments, is inadequate for evaluating the long-term effects of hydrogen-rich water on body composition, appetite regulation, or metabolic biomarkers, thereby limiting insights into sustained outcomes. Although biochemical markers such as GLP-1 and SCFA levels were measured, the study lacks detailed mechanistic exploration to elucidate direct pathways linking these markers to observed physiological changes. Furthermore, the exclusion of participants with chronic diseases, recent use of obesity-related pharmaceuticals, or dietary supplements narrows the study population, reducing its relevance to real-world scenarios where such conditions are prevalent among individuals with obesity. Future research addressing these limitations could significantly strengthen the robustness and external validity of findings related to hydrogen-rich water and its potential therapeutic benefits for obesity.

## 5. Conclusions

In conclusion, this study provides promising evidence that hydrogen-rich water may offer a safe and effective intervention for managing appetite, improving lipid profiles, and enhancing sleep quality in individuals with obesity. Over the eight-week intervention, participants consuming hydrogen-rich water showed significant reductions in food cravings, total and LDL cholesterol levels, and improvements in sleep quality compared to the placebo group. These effects were particularly pronounced in women, suggesting potential gender-specific responses. Although no major differences were observed in body composition or obesity-related quality of life, the findings support the need for further investigation into the long-term benefits and mechanistic pathways of hydrogen-rich water in obesity management.

## Figures and Tables

**Table 1:** Baseline characteristics of study participants (*n* = 36).

	Mean ± SD	Min–Max
Age (years)	42.1 ± 13.2	20–62
Female (%)	66.7	-
Weight (kg)	89.2 ± 17.6	61.6–141.1
Body mass index (kg/m^2^)	30.8 ± 4.2	24.0–42.1
Waist circumference (cm)	94.7 ± 11.6	74.0–122.5
Body fat percentage (%)	34.4 ± 7.0	25.0–47.5
Blood glucose (mmol/L)	5.47 ± 0.60	4.61–7.39
Total cholesterol (mmol/L)	5.33 ± 1.01	3.58–7.20
LDL cholesterol (mmol/L)	3.26 ± 0.90	1.87–5.44
HDL cholesterol (mmol/L)	1.46 ± 0.44	0.86–2.85
Triglycerides (mmol/L)	1.51 ± 1.43	0.59–8.67

**Table 2:** Food appetite domains in the experimental group (HRW) and control group (CON) at baseline and after 8 weeks. Values are expressed as mean ± SD.

	Group	Baseline	Follow-Up	Delta	95% CI	*p* ^†^	Cohen’s *d*	*p* ^‡^	*η_p_* ^2^
**Intense desire to eat**									
**Total**	HRW CON	12.9 ± 4.4 12.2 ± 3.4	11.2 ± 4.5 11.5 ± 2.9	1.8 ± 3.0 0.6 ± 3.4	0.3–3.3 −1.1–2.4	0.01 0.22	0.38 0.22	0.30	0.07
**Female**	HRW CON	13.7 ± 4.3 11.4 ± 3.9	11.5 ± 4.0 11.2 ± 3.1	2.2 ± 2.7 0.2 ± 4.1	0.5–3.9 −2.6–3.0	0.01 0.44	0.53 0.06	0.26	0.12
**Male**	HRW CON	11.3 ± 4.5 13.7 ± 1.6	10.3 ± 5.9 12.2 ± 2.5	1.0 ± 3.5 1.5 ± 1.4	−2.7–4.7 0.0–3.0	0.26 0.02	0.19 0.72	0.70	0.03
**Anticipation of positive reinforcement from eating**									
**Total**	HRW CON	11.8 ± 4.0 11.3 ± 3.2	10.6 ± 4.4 10.6 ± 2.4	1.2 ± 2.7 0.6 ± 2.5	−0.1–2.5 −0.7–1.9	0.04 0.15	0.28 0.25	0.64	0.01
**Female**	HRW CON	12.4 ± 3.8 10.1 ± 3.0	11.5 ± 4.3 10.2 ± 2.6	0.9 ± 3.1 −0.1 ± 2.4	−1.1–2.9 −1.7–1.5	0.16 0.45	0.22 0.04	0.53	0.04
**Male**	HRW CON	10.7 ± 4.3 13.5 ± 2.5	8.8 ± 4.4 11.5 ± 1.8	1.8 ± 1.8 2.0 ± 2.3	−0.1–3.7 −0.4–4.4	0.03 0.04	0.44 0.92	0.88	0.01
**Expectation of relief from negative emotional states through eating**									
**Total**	HRW CON	11.1 ± 4.4 9.5 ± 2.8	10.0 ± 3.5 9.6 ± 2.8	1.1 ± 2.1 −0.1 ± 2.5	0.1–2.1 −1.4–1.2	0.02 0.46	0.28 0.04	0.16	0.12
**Female**	HRW CON	11.6 ± 4.1 9.7 ± 2.9	10.4 ± 3.4 10.2 ± 2.7	1.2 ± 2.4 −0.5 ± 3.0	−0.3–2.7 −2.5–1.5	0.06 0.31	0.32 0.18	0.19	0.17
**Male**	HRW CON	10.3 ± 5.2 9.2 ± 2.9	9.3 ± 3.9 8.5 ± 3.0	1.0 ± 1.7 0.7 ± 1.4	−0.8–2.8 −0.8–2.2	0.10 0.14	0.22 0.24	0.66	0.04
**Lack of control over eating behaviors**									
**Total**	HRW CON	10.5 ± 4.1 10.0 ± 2.7	9.6 ± 4.1 9.8 ± 2.7	0.9 ± 2.8 0.2 ± 2.2	−0.5–2.3 −0.9–1.3	0.09 0.38	0.22 0.07	0.41	0.04
**Female**	HRW CON	11.3 ± 3.8 9.8 ± 3.1	10.1 ± 3.7 10.0 ± 3.1	1.2 + 3.1 −0.2 ± 1.9	−0.8–3.2 −1.5–1.1	0.11 0.38	0.32 0.07	0.17	0.18
**Male**	HRW CON	9.0 ± 4.6 10.3 ± 2.1	8.5 ± 4.9 9.5 ± 2.1	0.5 ± 2.4 0.8 ± 2.9	−2.0–3.0 −2.2–3.8	0.32 0.25	0.11 0.38	0.82	0.01
**Cravings as a physiological state**									
**Total**	HRW CON	11.8 ± 2.9 10.4 ± 3.6	9.5 ± 4.3 10.5 ± 2.6	2.3 ± 3.3 −0.1 ± 3.2	0.7–3.9 −1.7–1.5	<0.01 0.47	0.63 0.03	0.05	0.22
**Female**	HRW CON	11.7 ± 3.0 10.4 ± 3.8	9.2 ± 3.7 10.9 ± 2.8	2.5 ± 3.3 −0.5 ± 3.7	0.4–4.6 −3.0–2.0	0.01 0.32	0.74 0.15	0.05	0.33
**Male**	HRW CON	11.8 ± 3.1 10.5 ± 3.5	10.0 ± 5.6 9.7 ± 2.3	1.8 ± 3.6 0.8 ± 1.8	−2.0–5.6 −1.1–2.7	0.13 0.16	0.40 0.27	0.47	0.11
**Total score**									
**Total**	HRW CON	58.2 ± 17.1 53.3 ± 12.7	50.8 ± 18.9 52.0 ± 11.0	7.4 ± 5.6 1.3 ± 10.0	4.6–10.2 −3.8–6.4	<0.01 0.29	0.41 0.11	0.05	0.21
**Female**	HRW CON	60.7 ± 16.0 51.3 ± 14.1	52.7 ± 17.1 52.4 ± 11.8	8.0 ± 4.5 −1.1 ± 11.0	5.1–10.9 −8.5–6.3	<0.01 0.37	0.48 0.09	0.03	0.41
**Male**	HRW CON	53.1 ± 19.6 57.1 ± 9.6	46.9 ± 23.5 51.3 ± 10.1	6.2 ± 7.8 5.8 ± 6.4	−2.0–14.4 −0.9–12.5	0.06 0.04	0.29 0.59	0.89	0.01

*Abbreviations*: HRW, hydrogen-rich water; CON, control water. The dagger (†) indicates statistical significance for within-group comparison versus baseline levels. The double dagger (‡) indicates statistical significance for interaction effect (time vs. treatment).

**Table 3:** Body size and body composition in the experimental group (HRW) and control group (CON) at baseline and after 8 weeks. Values are expressed as mean ± SD.

	Group	Baseline	Follow-Up	Delta	95% CI	*p* ^†^	Cohen’s *d*	*p* ^‡^	*η_p_* ^2^
**Weight (kg)**									
**Total**	HRW CON	90.0 ± 19.3 88.3 ± 16.5	89.4 ± 19.0 88.2 ± 17.1	0.6 ± 2.0 0.0 ± 1.3	−0.4–1.6 −0.7–0.7	0.26 0.46	0.03 0.01	0.41	0.04
**Female**	HRW CON	81.7 ± 13.5 79.8 ± 13.6	81.8 ± 13.2 79.7 ± 13.9	−0.2 ± 1.4 0.1 ± 1.5	−1.1–0.7 −0.9–1.1	0.36 0.44	0.01 0.01	0.77	0.01
**Male**	HRW CON	106.7 ± 19.1 103.9 ± 9.2	104.7 ± 20.6 103.9 ± 9.4	2.0 ± 2.4 0.0 ± 0.7	−0.5–4.5 −0.7–0.7	0.05 0.44	0.10 0.00	0.07	0.52
**Body mass index (kg/m^2^)**									
**Total**	HRW CON	30.9 ± 4.3 30.4 ± 4.5	30.9 ± 4.4 30.2 ± 3.9	0.0 ± 0.8 0.2 ± 1.1	−0.4–0.4 −0.4–0.8	0.49 0.21	0.01 0.05	0.34	0.06
**Female**	HRW CON	30.4 ± 3.8 29.5 ± 5.3	30.5 ± 3.8 29.1 ± 4.3	−0.1 ± 0.7 0.3 ± 1.3	−0.5–0.3 −0.6–1.2	0.26 0.22	0.03 0.08	0.44	0.06
**Male**	HRW CON	32.0 ± 5.2 32.1 ± 2.2	31.7 ± 5.7 32.0 ± 2.3	0.3 ± 1.1 0.0 ± 0.4	−0.9–1.5 −0.4–0.4	0.27 0.41	0.06 0.04	0.54	0.08
**Waist circumference (cm)**									
**Total**	HRW CON	96.0 ± 12.5 93.4 ± 11.2	96.0 ± 12.7 93.3 ± 11.6	0.0 ± 3.0 0.1 ± 2.4	−1.5–1.5 −1.1–1.3	0.49 0.46	0.01 0.06	0.82	0.01
**Female**	HRW CON	91.2 ± 10.9 88.6 ± 9.7	91.5 ± 11.2 88.8 ± 9.3	−0.3 ± 2.3 −0.2 ± 2.0	−1.8–1.2 −1.5–1.1	0.35 0.39	0.03 0.02	0.82	0.01
**Male**	HRW CON	105.4 ± 10.2 102.0 ± 9.2	105.0 ± 11.5 101.5 ± 11.5	0.5 ± 4.4 0.5 ± 3.2	−4.1–5.1 −2.9–3.9	0.41 0.36	0.04 0.05	0.99	0.00
**Fat mass (%)**									
**Total**	HRW CON	35.0 ± 6.9 33.2 ± 7.2	34.6 ± 8.6 33.4 ± 6.8	0.4 ± 3.5 −0.2 ± 2.3	−1.3–2.1 −1.4–1.0	0.31 0.40	0.05 0.02	0.53	0.03
**Female**	HRW CON	38.1 ± 4.4 36.8 ± 5.2	38.2 ± 6.3 36.7 ± 4.8	0.0 ± 3.7 0.1 ± 2.1	−2.4–2.4 −1.3–1.5	0.48 0.45	0.02 0.02	0.46	0.06
**Male**	HRW CON	28.8 ± 7.0 26.7 ± 5.1	27.5 ± 8.6 27.2 ± 5.7	1.3 ± 3.0 −0.6 ± 2.9	−1.8–4.4 −3.6–2.4	0.17 0.33	0.17 0.09	0.23	0.27
**Fat free mass (kg)**									
**Total**	HRW CON	60.0 ± 13.5 59.2 ± 14.3	59.8 ± 13.3 58.9 ± 14.1	0.2 ± 2.9 0.3 ± 2.4	−1.2–1.6 −0.9–1.5	0.38 0.30	0.02 0.02	0.74	0.01
**Female**	HRW CON	52.5 ± 8.6 49.8 ± 6.0	53.2 ± 7.4 49.8 ± 6.3	0.2 ± 3.3 0.0 ± 1.5	−1.9–2.3 −1.0–1.0	0.40 0.46	0.09 0.00	0.50	0.05
**Male**	HRW CON	75.0 ± 7.2 76.3 ± 6.5	74.9 ± 8.7 75.4 ± 6.6	0.1 ± 2.3 0.9 ± 3.6	−2.3–2.5 −2.9–4.7	0.44 0.27	0.01 0.14	0.60	0.06
**Muscle mass (kg)**									
**Total**	HRW CON	26.8 ± 8.2 27.2 ± 8.4	26.7 ± 8.2 27.1 ± 8.2	0.1 ± 1.1 0.1 ± 1.0	−0.4–0.6 −0.4–0.6	0.41 0.30	0.10 0.01	0.70	0.01
**Female**	HRW CON	21.5 ± 2.9 21.5 ± 2.6	21.5 ± 2.1 21.6 ± 2.8	0.0 ± 1.3 −0.1 ± 0.5	−0.8–0.8 −0.4–0.2	0.46 0.32	0.00 0.04	0.51	0.05
**Male**	HRW CON	37.3 ± 3.7 37.7 ± 3.1	37.2 ± 4.3 37.2 ± 3.3	0.1 ± 0.8 0.5 ± 1.5	−0.7–0.9 −1.2–2.1	0.39 0.23	0.03 0.16	0.52	0.09
**Total body water (L)**									
**Total**	HRW CON	45.6 ± 10.3 45.7 ± 10.6	45.3 ± 9.4 45.4 ± 10.5	0.3 ± 3.6 0.3 ± 2.7	−1.5–2.1 −1.1–1.7	0.36 0.36	0.03 0.02	0.97	0.01
**Female**	HRW CON	40.3 ± 7.6 38.9 ± 5.5	39.8 ± 4.3 39.0 ± 6.0	0.6 ± 4.2 −0.1 ± 1.5	−2.1–3.1 −1.1–0.9	0.32 0.39	0.08 0.02	0.59	0.03
**Male**	HRW CON	56.1 ± 5.9 58.0 ± 5.0	56.3 ± 6.6 57.1 ± 5.0	−0.2 ± 2.2 0.5 ± 3.6	−2.5–2.1 −3.3–4.3	0.41 0.38	0.03 0.18	0.58	0.07
**Intracellular water (L)**									
**Total**	HRW CON	25.3 ± 6.5 25.3 ± 6.9	25.3 ± 6.2 25.5 ± 6.7	0.1 ± 2.2 −0.2 ± 2.5	−1.0–1.2 −1.5–1.1	0.45 0.38	0.01 0.03	0.78	0.01
**Female**	HRW CON	21.7 ± 4.3 20.7 ± 3.1	21.5 ± 2.5 21.4 ± 3.6	0.3 ± 2.5 −0.7 ± 2.0	−1.3–1.9 −2.0–0.6	0.35 0.15	0.06 0.21	0.69	0.02
**Male**	HRW CON	32.5 ± 3.2 33.9 ± 3.1	32.9 ± 3.7 33.2 ± 3.2	−0.4 ± 1.4 0.7 ± 3.2	−1.9–1.1 −2.7–4.1	0.28 0.31	0.11 0.22	0.52	0.09
**Extracellular water (L)**									
**Total**	HRW CON	20.3 ± 3.9 20.1 ± 3.7	20.0 ± 3.3 20.0 ± 3.7	0.2 ± 1.5 0.1 ± 0.9	−0.5–0.9 −0.4–0.6	0.25 0.28	0.07 0.04	0.98	0.01
**Female**	HRW CON	18.6 ± 3.3 17.9 ± 2.3	18.3 ± 1.9 17.8 ± 2.5	0.3 ± 1.7 0.1 ± 0.8	−0.8–1.4 −0.4–0.6	0.28 0.38	0.11 0.04	0.61	0.03
**Male**	HRW CON	23.5 ± 2.8 24.2 ± 2.0	23.4 ± 3.0 23.9 ± 1.9	0.1 ± 1.2 0.2 ± 1.2	−1.2–1.4 −1.1–1.5	0.39 0.33	0.03 0.15	0.87	0.01
**Protein mass (L)**									
**Total**	HRW CON	9.4 ± 3.8 9.8 ± 3.5	9.5 ± 3.5 9.7 ± 3.4	−0.1 ± 0.8 0.2 ± 1.0	−0.5–0.3 −0.3–0.7	0.28 0.27	0.03 0.04	0.33	0.06
**Female**	HRW CON	7.0 ± 1.5 7.7 ± 1.9	7.3 ± 1.0 7.5 ± 1.7	−0.3 ± 0.8 0.2 ± 1.1	−0.8–0.2 −0.5–0.9	0.12 0.30	0.24 0.11	0.50	0.05
**Male**	HRW CON	14.0 ± 2.1 13.6 ± 1.8	13.8 ± 2.7 13.5 ± 1.7	0.3 ± 0.7 0.1 ± 0.6	−0.4–1.0 −0.5–0.7	0.19 0.38	0.08 0.06	0.77	0.02
**Mineral mass (L)**									
**Total**	HRW CON	3.6 ± 1.2 3.7 ± 1.1	3.6 ± 1.1 3.7 ± 1.0	−0.1 ± 0.3 0.1 ± 0.4	−0.2–0.0 −0.1–0.3	0.26 0.28	0.04 0.05	0.32	0.06
**Female**	HRW CON	2.9 ± 0.6 3.2 ± 0.8	3.0 ± 0.4 3.1 ± 0.7	−0.1 ± 0.3 0.1 ± 0.5	−0.3–0.1 −0.2–0.4	0.12 0.28	0.20 0.13	0.37	0.08
**Male**	HRW CON	4.9 ± 0.8 4.8 ± 0.6	4.8 ± 1.0 4.8 ± 0.6	0.1 ± 0.2 0.0 ± 0.2	−0.1–0.3 −0.2–0.2	0.19 0.48	0.11 0.00	0.50	0.10
**Total body calcium (kg)**									
**Total**	HRW CON	1.2 ± 0.3 1.2 ± 0.3	1.2 ± 0.3 1.2 ± 0.3	0.0 ± 0.1 0.0 ± 0.5	0.0–0.0 −0.3–0.3	0.39 0.38	0.01 0.01	0.94	0.01
**Female**	HRW CON	1.0 ± 0.1 1.0 ± 0.1	1.0 ± 0.1 1.0 ± 0.1	0.0 ± 0.1 0.0 ± 0.3	−0.1–0.1 −0.2–0.2	0.37 0.27	0.00 0.00	0.72	0.01
**Male**	HRW CON	1.6 ± 0.2 1.6 ± 0.1	1.6 ± 0.2 1.6 ± 0.2	0.0 ± 0.1 0.0 ± 0.1	−0.1–0.1 −0.1–0.1	0.47 0.27	0.00 0.00	0.53	0.08
**Glycogen mass (kg)**									
**Total**	HRW CON	531 ± 126 538 ± 129	530 ± 123 534 ± 128	1.3 ± 25.9 3.7 ± 21.6	−11.6–14.2 −7.4–14.8	0.41 0.24	0.01 0.03	0.60	0.02
**Female**	HRW CON	456 ± 64 454 ± 52	455 ± 45 453 ± 58	1.3 ± 28.8 1.0 ± 13.7	−17.0–19.6 −8.2–10.2	0.44 0.41	0.02 0.02	0.39	0.07
**Male**	HRW CON	682 ± 66 694 ± 59	680 ± 79 685 ± 60	1.3 ± 21.1 8.7 ± 32.7	−20.8–23.4 −25.6–43.0	0.44 0.27	0.03 0.15	0.60	0.06

*Abbreviations*: HRW, hydrogen-rich water; CON, control water. The dagger (†) indicates statistical significance for within-group comparison versus baseline levels. The double dagger (‡) indicates statistical significance for interaction effect (time vs. treatment).

**Table 4:** Obesity-related quality of life in the experimental group (HRW) and control group (CON) at the baseline and after 8 weeks. Values are expressed as mean ± SD.

	Group	Baseline	Follow-Up	Delta	95% CI	*p* ^†^	Cohen’s *d*	*p* ^‡^	*η_p_* ^2^
**Physical function (score)**									
**Total**	HRW CON	70.2 ± 16.2 79.4 ± 24.0	77.2 ± 14.3 82.5 ± 25.9	−7.0 ± 13.0 −3.1 ± 9.2	−13.5–−0.5 −7.8–1.6	0.02 0.09	0.46 0.12	0.30	0.07
**Female**	HRW CON	68.9 ± 17.5 75.4 ± 28.0	78.3 ± 15.6 80.8 ± 28.9	−9.4 ± 12.2 −5.4 ± 8.6	−17.2–−1.6 −11.2–0.4	0.01 0.03	0.57 0.19	0.42	0.07
**Male**	HRW CON	72.7 ± 14.2 86.7 ± 14.0	75.0 ± 12.2 85.6 ± 21.5	−2.3 ± 14.3 1.1 ± 9.5	−17.3–12.7 −8.9–11.1	0.36 0.39	0.17 0.06	0.61	0.06
**Self-esteem (score)**									
**Total**	HRW CON	68.7 ± 28.4 67.0 ± 27.4	72.8 ± 22.5 72.5 ± 27.5	−4.1 ± 10.3 −5.5 ± 8.8	−9.2–1.0 −10.0–−1.0	0.05 0.01	0.16 0.20	0.67	0.01
**Female**	HRW CON	62.5 ± 31.7 64.0 ± 31.2	69.6 ± 26.4 67.9 ± 32.2	−7.1 ± 10.1 −3.9 ± 8.7	−13.5–−0.7 −9.7–1.9	0.02 0.08	0.24 0.12	0.14	0.05
**Male**	HRW CON	81.0 ± 15.8 72.6 ± 20.9	79.1 ± 11.3 81.0 ± 14.9	1.9 ± 8.6 −8.3 ± 8.9	−7.1–10.9 −17.6–1.0	0.31 0.04	0.14 0.46	<0.01	0.87
**Sexual life (score)**									
**Total**	HRW CON	78.8 ± 21.7 88.6 ± 26.3	84.0 ± 19.3 91.5 ± 24.7	−5.2 ± 12.0 −2.9 ± 14.2	−11.2–0.8 −10.2–4.4	0.04 0.20	0.25 0.11	0.51	0.03
**Female**	HRW CON	74.5 ± 22.2 82.4 ± 31.5	81.8 ± 21.4 86.9 ± 30.2	−7.3 ± 13.8 −4.5 ± 17.7	−16.1–1.5 −16.4–7.4	0.05 0.21	0.34 0.15	0.34	0.03
**Male**	HRW CON	87.5 ± 19.4 100.0 ± 0.0	88.5 ± 15.0 100.0 ± 0.0	−1.0 ± 6.1 0.0 ± 0.0	−7.4–5.4 0.0–0.0	0.35 0.99	0.06 0.00	0.69	0.03
**Public distress (score)**									
**Total**	HRW CON	95.3 ± 7.8 92.1 ± 22.5	95.6 ± 9.8 92.4 ± 24.0	−0.3 ± 4.0 −0.3 ± 4.5	−2.3–1.7 −2.1–1.5	0.39 0.40	0.03 0.01	0.99	0.01
**Female**	HRW CON	95.0 ± 9.0 89.1 ± 28.2	95.0 ± 11.9 89.5 ± 29.8	0.0 ± 4.8 −0.5 ± 5.2	−3.0–3.0 −4.0–3.0	0.50 0.39	0.00 0.01	0.99	0.02
**Male**	HRW CON	95.8 ± 4.9 97.5 ± 6.1	96.7 ± 4.1 97.5 ± 4.2	−0.8 ± 2.0 0.0 ± 3.2	−2.9–1.3 −3.4–3.4	0.18 0.50	0.20 0.00	0.61	0.06
**Work (score)**									
**Total**	HRW CON	94.4 ± 9.3 91.5 ± 24.8	91.3 ± 10.9 91.9 ± 24.1	3.1 ± 11.0 −0.4 ± 4.6	−2.4–8.6 −2.8–2.0	0.12 0.36	0.31 0.02	0.18	0.11
**Female**	HRW CON	97.9 ± 4.1 88.1 ± 31.1	90.6 ± 11.5 89.2 ± 29.9	7.3 ± 9.5 −1.1 ± 3.8	1.3–13.3 −3.7–1.5	0.01 0.17	0.85 0.04	0.57	0.37
**Male**	HRW CON	87.5 ± 13.1 97.9 ± 3.2	92.7 ± 7.3 97.0 ± 5.1	−5.2 ± 9.2 1.0 ± 6.0	−14.9–4.5 −5.3–7.3	0.11 0.35	0.49 0.21	0.18	0.33
**Total scores (score)**									
**Total**	HRW CON	80.9 ± 13.1 83.7 ± 22.9	84.1 ± 12.5 86.2 ± 23.2	−3.2 ± 5.4 −2.4 ± 5.2	−5.9–−0.5 −5.1–0.3	0.01 0.04	0.25 0.11	0.69	0.01
**Female**	HRW CON	78.9 ± 14.1 79.8 ± 28.0	82.9 ± 14.3 82.9 ± 28.4	−4.1 ± 5.8 −3.1 ± 6.3	−7.8–−0.4 −7.2–1.1	0.02 0.07	0.28 0.11	0.66	0.02
**Male**	HRW CON	84.9 ± 10.7 91.0 ± 6.7	86.4 ± 8.5 92.2 ± 6.5	−1.5 ± 4.6 −1.2 ± 2.3	−6.3–3.3 −3.6–1.2	0.23 0.12	0.16 0.18	0.88	0.01

*Abbreviations*: HRW, hydrogen-rich water; CON, control water. The dagger (†) indicates statistical significance for within-group comparison versus baseline levels. The double dagger (‡) indicates statistical significance for interaction effect (time vs. treatment).

**Table 5:** Sleep quality indices in the experimental group (HRW) and control group (CON) at the baseline and after 8 weeks. Values are expressed as mean ± SD.

	Group	Baseline	Follow-Up	Delta	95% CI	*p* ^†^	Cohen’s *d*	*p* ^‡^	*η_p_* ^2^
**Subjective sleep quality**									
**Total**	HRW CON	1.7 ± 0.8 1.8 ± 0.9	0.7 ± 0.7 1.2 ± 0.7	1.0 ± 0.8 0.6 ± 0.8	0.6–1.4 0.2–1.0	<0.01 <0.01	1.33 0.74	0.05	0.21
**Female**	HRW CON	1.8 ± 0.7 1.7 ± 0.8	0.6 ± 0.7 1.0 ± 0.6	1.3 ± 0.9 0.7 ± 0.6	0.7–1.9 0.3–1.1	<0.01 <0.01	1.71 0.99	0.14	0.21
**Male**	HRW CON	1.5 ± 0.8 1.8 ± 1.2	1.0 ± 0.6 1.5 ± 0.5	0.5 ± 0.5 0.3 ± 1.0	0.0–1.0 −0.7–1.3	0.04 0.23	0.71 0.45	0.61	0.06
**Sleep latency**									
**Total**	HRW CON	1.6 ± 1.4 1.5 ± 1.3	0.9 ± 1.3 0.7 ± 0.9	0.7 ± 1.6 0.8 ± 1.1	−0.1–1.5 0.2–1.4	0.05 0.01	0.52 0.72	0.91	0.00
**Female**	HRW CON	1.9 ± 1.5 1.3 ± 0.8	0.8 ± 1.3 0.5 ± 0.7	1.1 ± 1.6 0.7 ± 0.8	0.1–2.1 0.2–1.2	0.02 0.01	0.78 1.06	0.64	0.02
**Male**	HRW CON	1.0 ± 1.1 1.8 ± 1.8	1.3 ± 1.0 1.0 ± 1.3	−0.3 ± 1.4 0.8 ± 1.7	−1.9–1.3 −1.0–2.6	0.29 0.14	0.29 0.51	0.34	0.18
**Sleep duration**									
**Total**	HRW CON	0.8 ± 0.8 1.1 ± 1.2	0.8 ± 0.6 0.8 ± 0.9	−0.1 ± 0.9 0.4 ± 0.7	−0.5–0.3 0.0–0.8	0.40 0.03	0.00 0.28	0.07	0.20
**Female**	HRW CON	0.9 ± 0.9 1.2 ± 1.1	0.7 ± 0.7 0.6 ± 0.5	0.3 ± 1.0 0.5 ± 0.8	−0.3–0.9 0.0–1.0	0.19 0.03	0.25 0.70	0.52	0.04
**Male**	HRW CON	0.5 ± 0.5 0.8 ± 1.3	1.2 ± 0.4 1.0 ± 1.4	−0.7 ± 0.5 0.2 ± 0.5	−1.2–−0.2 −0.3–0.7	0.01 0.99	1.54 0.15	0.07	0.60
**Sleep efficiency**									
**Total**	HRW CON	0.1 ± 0.2 0.4 ± 0.8	0.1 ± 0.3 0.5 ± 0.8	−0.1 ± 0.5 −0.1 ± 0.6	−0.3–0.1 −0.4–0.2	0.29 0.22	0.00 0.13	0.77	0.01
**Female**	HRW CON	0.1 ± 0.3 0.2 ± 0.3	0.2 ± 0.4 0.2 ± 0.4	0.1 ± 0.5 0.0 ± 0.6	−0.2–0.4 −0.4–0.4	0.29 0.50	0.28 0.00	0.99	0.00
**Male**	HRW CON	0.0 ± 0.0 0.7 ± 1.2	0.0 ± 0.0 1.2 ± 1.1	0.0 ± 0.0 −0.3 ± 0.5	0.0–0.0 −0.8–0.2	0.99 0.09	0.00 0.43	0.18	0.40
**Sleep disturbance**									
**Total**	HRW CON	1.2 ± 0.5 1.3 ± 0.8	0.9 ± 0.5 1.2 ± 0.6	0.2 ± 0.5 0.1 ± 0.5	0.0–0.4 −0.2–0.4	0.05 0.17	0.60 0.14	0.54	0.02
**Female**	HRW CON	1.3 ± 0.5 1.3 ± 0.7	1.1 ± 0.5 1.3 ± 0.6	0.3 ± 0.6 0.0 ± 0.4	−0.1–0.7 −0.3–0.3	0.10 0.50	0.40 0.00	0.19	0.16
**Male**	HRW CON	0.8 ± 0.4 1.3 ± 1.0	0.7 ± 0.5 1.0 ± 0.6	0.2 ± 0.4 0.3 ± 0.5	−0.2–0.6 −0.2–0.8	0.18 0.09	0.22 0.36	0.61	0.06
**Use of sleep medication**									
**Total**	HRW CON	0.2 ± 0.7 0.4 ± 1.0	0.5 ± 1.0 0.4 ± 0.9	−0.3 ± 0.8 0.0 ± 0.6	−0.7–0.1 −0.3–0.3	0.07 0.50	0.35 0.00	0.24	0.09
**Female**	HRW CON	0.3 ± 0.9 0.4 ± 1.0	0.6 ± 1.0 0.3 ± 0.9	−0.3 ± 0.7 0.1 ± 0.3	−0.7–0.1 −0.1–0.3	0.05 0.17	0.32 0.11	0.05	0.33
**Male**	HRW CON	0.2 ± 0.4 0.5 ± 1.2	0.2 ± 0.4 0.7 ± 1.0	0.0 ± 0.0 −0.2 ± 1.0	0.0–0.0 −1.2–0.8	0.99 0.35	0.00 0.18	0.70	0.03
**Daytime dysfunction**									
**Total**	HRW CON	1.4 ± 0.7 1.1 ± 1.1	0.8 ± 0.5 0.6 ± 0.9	0.6 ± 0.9 0.5 ± 0.9	0.2–1.0 0.1–0.9	0.01 0.02	0.99 0.50	0.71	0.01
**Female**	HRW CON	1.4 ± 0.8 1.0 ± 1.1	0.8 ± 0.6 0.5 ± 0.9	0.6 ± 0.9 0.5 ± 1.1	0.0–1.2 −0.2–1.2	0.02 0.11	0.85 0.50	0.84	0.00
**Male**	HRW CON	1.3 ± 0.5 1.3 ± 1.2	0.7 ± 0.5 0.7 ± 0.8	0.7 ± 1.0 0.7 ± 0.5	−0.3–1.7 0.2–1.2	0.09 0.01	1.20 0.59	0.99	0.00
**Total score**									
**Total**	HRW CON	6.9 ± 2.5 7.5 ± 4.9	4.8 ± 2.2 5.2 ± 4.1	2.2 ± 3.3 2.2 ± 2.5	0.6–3.8 0.9–3.5	0.01 <0.01	0.89 0.51	0.92	0.00
**Female**	HRW CON	7.8 ± 2.6 7.0 ± 3.2	4.8 ± 2.6 4.5 ± 3.1	3.0 ± 3.7 2.5 ± 2.1	0.6–5.4 1.1–3.9	0.01 <0.01	1.15 0.79	0.77	0.01
**Male**	HRW CON	5.3 ± 1.2 8.3 ± 7.3	4.8 ± 1.3 6.7 ± 5.6	0.5 ± 1.6 1.7 ± 3.3	−1.2–2.2 −1.8–5.2	0.24 0.14	0.40 0.25	0.51	0.09

*Abbreviations*: HRW, hydrogen-rich water; CON, control water. The dagger (†) indicates statistical significance for within-group comparison versus baseline levels. The double dagger (‡) indicates statistical significance for interaction effect (time vs. treatment).

**Table 6:** Biochemical markers in the experimental group (HRW) and control group (CON) at the baseline and after 8 weeks. Values are expressed as mean ± SD.

	Group	Baseline	Follow-Up	Delta	95% CI	*p* ^†^	Cohen’s *d*	*p* ^‡^	*η_p_* ^2^
**GLP-1 (pg/mL)**									
**Total**	HRW CON	69.3 ± 50.7 80.6 ± 70.5	86.2 ± 65.4 78.1 ± 51.2	−16.9 ± 40.1 2.4 ± 37.0	−36.8–3.0 −16.6–21.4	0.05 0.40	0.29 0.04	0.05	0.20
**Female**	HRW CON	60.9 ± 47.3 70.2 ± 75.6	88.5 ± 70.7 74.2 ± 61.2	−27.6 ± 43.2 −4.0 ± 30.4	−55.0–−0.2 −16.4–24.4	0.02 0.34	0.36 0.21	0.09	0.25
**Male**	HRW CON	86.6 ± 56.7 99.6 ± 61.7	81.4 ± 59.7 85.4 ± 28.5	5.2 ± 22.2 14.1 ± 47.8	−18.1–28.5 −36.1–64.3	0.30 0.25	0.09 0.29	0.58	0.07
**Acetic acid (µg/mL)**									
**Total**	HRW CON	3.35 ± 1.01 3.11 ± 0.72	3.03 ± 0.85 2.76 ± 0.64	0.32 ± 1.05 0.35 ± 0.90	−0.31–0.95 −0.09–0.79	0.11 0.06	0.34 0.52	0.95	<0.01
**Female**	HRW CON	3.12 ± 0.76 3.10 ± 0.52	2.73 ± 0.52 2.87 ± 0.47	0.39 ± 0.92 0.23 ± 0.70	−0.16–0.94 −0.21–0.67	0.09 0.16	0.60 0.46	0.65	0.02
**Male**	HRW CON	3.81 ± 1.34 3.14 ± 1.06	3.63 ± 1.11 2.55 ± 0.90	0.18 ± 1.35 0.59 ± 1.23	−1.30–1.76	0.38 0.15	0.14 0.60	0.43	0.13
**Propionic acid (µg/mL)**									
**Total**	HRW CON	0.37 ± 0.20 0.31 ± 0.07	0.23 ± 0.05 0.22 ± 0.05	0.14 ± 0.20 0.09 ± 0.09	0.04–0.24 0.05–0.13	0.01 <0.01	0.91 1.51	0.30	0.07
**Female**	HRW CON	0.30 ± 0.09 0.31 ± 0.06	0.23 ± 0.05 0.23 ± 0.03	0.07 ± 0.11 0.08 ± 0.08	0.01–0.13 0.04–0.12	0.02 <0.01	0.99 1.64	0.89	<0.01
**Male**	HRW CON	0.50 ± 0.31 0.32 ± 0.09	0.24 ± 0.03 0.21 ± 0.07	0.26 ± 0.30 0.11 ± 0.11	−0.02–0.54 0.01–0.21	0.04 0.03	1.20 1.35	0.20	0.31
**Butyric acid (µg/mL)**									
**Total**	HRW CON	1.52 ± 0.49 1.42 ± 0.45	1.03 ± 0.29 0.97 ± 0.26	0.49 ± 0.48 0.45 ± 0.56	0.22–0.76 0.19–0.71	<0.01 <0.01	1.96 1.22	0.72	0.01
**Female**	HRW CON	1.45 ± 0.26 1.39 ± 0.31	1.10 ± 0.30 1.00 ± 0.27	0.35 ± 0.29 0.39 ± 0.42	0.11–0.59 0.13–0.65	<0.01 0.01	1.60 1.36	0.91	<0.01
**Male**	HRW CON	1.66 ± 0.79 1.48 ± 0.67	0.90 ± 0.23 0.93 ± 0.25	0.75 ± 0.69 0.55 ± 0.81	0.01–1.51 −0.10–1.20	0.02 0.08	2.79 1.09	0.58	0.07
**Breath hydrogen (ppm)**									
**Total**	HRW CON	34 ± 29 29 ± 31	28 ± 30 26 ± 26	6 ± 45 3 ± 42	−16–28 −19–25	0.28 0.38	0.21 0.10	0.76	0.01
**Female**	HRW CON	36 ± 32 21 ± 24	23 ± 18 27 ± 32	14 ± 41 6 ± 38	−12–40 −20–32	0.14 0.31	0.52 0.21	0.26	0.13
**Male**	HRW CON	30 ± 24 44 ± 38	39 ± 45 24 ± 15	9 ± 52 20 ± 47	−46–64 −29–69	0.35 0.18	0.24 0.67	0.47	0.11
**Glucose (mmol/L)**									
**Total**	HRW CON	5.60 ± 0.56 5.38 ± 0.66	5.63 ± 0.66 5.46 ± 0.96	−0.03 ± 0.42 −0.09 ± 0.47	−0.24–0.18 −0.33–0.15	0.39 0.23	0.05 0.10	0.87	<0.01
**Female**	HRW CON	5.60 ± 0.63 5.28 ± 0.81	5.64 ± 0.77 5.50 ± 1.16	−0.05 ± 0.50 −0.22 ± 0.49	−0.37–0.27 −0.55–0.11	0.38 0.08	0.07 0.22	0.43	0.07
**Male**	HRW CON	5.60 ± 0.43 5.55 ± 0.25	5.60 ± 0.39 5.40 ± 0.46	0.00 ± 0.24 0.16 ± 0.34	−0.25–0.25 −0.20–0.52	0.49 0.16	0.01 0.42	0.39	0.15
**Total cholesterol (mmol/L)**									
**Total**	HRW CON	5.63 ± 1.14 5.05 ± 0.79	5.31 ± 1.13 5.16 ± 0.86	0.32 ± 0.44 −0.11 ± 0.55	0.10–0.54 −0.39–0.17	<0.01 0.20	0.28 0.14	0.02	0.31
**Female**	HRW CON	5.49 ± 1.23 5.08 ± 0.69	5.27 ± 1.20 5.37 ± 0.73	0.21 ± 0.46 −0.29 ± 0.56	−0.08–0.50 −0.67–0.09	0.07 0.06	0.18 0.40	0.04	0.35
**Male**	HRW CON	5.91 ± 0.96 5.00 ± 0.99	5.39 ± 1.06 4.79 ± 1.03	0.52 ± 0.37 0.21 ± 0.38	0.13–0.91 −0.19–0.61	0.01 0.12	0.51 0.20	0.16	0.35
**LDL cholesterol (mmol/L)**									
**Total**	HRW CON	3.45 ± 1.06 3.08 ± 0.73	3.16 ± 1.03 3.15 ± 0.61	0.28 ± 0.53 −0.07 ± 0.48	0.02–0.54 −0.32–0.18	0.02 0.29	0.28 0.10	0.04	0.24
**Female**	HRW CON	3.21 ± 0.99 3.02 ± 0.69	3.02 ± 0.98 3.26 ± 0.56	0.20 ± 0.52 −0.21 ± 0.47	−0.13–0.53 −0.53–0.11	0.11 0.08	0.20 0.38	0.08	0.30
**Male**	HRW CON	4.04 ± 1.11 3.18 ± 0.85	3.45 ± 1.17 2.96 ± 0.71	0.49 ± 0.57 0.22 ± 0.32	−0.11–1.09 −0.12–0.56	0.06 0.08	0.52 0.28	0.30	0.26
**HDL cholesterol (mmol/L)**									
**Total**	HRW CON	1.57 ± 0.52 1.34 ± 0.31	1.51 ± 0.52 1.32 ± 0.34	0.07 ± 0.15 0.02 ± 0.14	0.00–0.14 −0.05–0.09	0.04 0.29	0.13 0.06	0.30	0.07
**Female**	HRW CON	1.74 ± 0.56 1.40 ± 0.36	1.65 ± 0.57 1.39 ± 0.39	0.09 ± 0.14 0.00 ± 0.16	0.00–0.18 −0.11–0.11	0.02 0.46	0.16 0.01	0.30	0.11
**Male**	HRW CON	1.24 ± 0.07 1.24 ± 0.19	1.22 ± 0.20 1.19 ± 0.17	0.02 ± 0.18 0.05 ± 0.13	−01.7–0.21 −0.09–0.19	0.41 0.21	0.12 0.26	0.68	0.04
**Triglycerides (mmol/L)**									
**Total**	HRW CON	1.41 ± 0.85 1.63 ± 1.95	1.43 ± 0.63 1.92 ± 2.58	−0.02 ± 0.67 −0.28 ± 0.76	−0.35–0.31 −0.67–0.11	0.46 0.07	0.02 0.12	0.46	0.04
**Female**	HRW CON	1.17 ± 0.20 1.83 ± 2.46	1.35 ± 0.62 2.19 ± 3.19	−0.18 ± 0.51 −0.37 ± 0.91	−0.50–0.14 −0.98–0.24	0.12 0.11	0.39 0.13	0.43	0.06
**Male**	HRW CON	1.90 ± 1.40 1.28 ± 0.45	1.59 ± 0.67 1.41 ± 0.61	0.31 ± 0.87 −0.13 ± 0.34	−0.60–1.22 −0.49–0.23	0.21 0.20	0.28 0.24	0.13	0.40

*Abbreviations*: HRW, hydrogen-rich water; CON, control water; GLP-1, glucagon-like peptide-1. The dagger (†) indicates statistical significance for within-group comparison versus baseline levels. The double dagger (‡) indicates statistical significance for interaction effect (time vs. treatment).

## Data Availability

All data analyzed are included in the article. Further inquiries can be directed to the corresponding author.
